# Diagnosis and Management of Patients with Patent Foramen Ovale: Current Evidence and Future Perspective

**DOI:** 10.3390/jcdd13080359

**Published:** 2026-08-01

**Authors:** Tiziana Formisano, Roberta Bottino, Saverio D’Elia, Rosa Franzese, Daniele Molinari, Andreina Carbone, Pasquale Castaldo, Massimiliano Orlandi, Simona Sperlongano, Alberto Palladino, Consiglia Barbareschi, Giovanni Cimmino

**Affiliations:** 1 Cardiology Unit, Azienda Ospedaliera Universitaria Luigi Vanvitelli, 80138 Naples, Italy; tiziana.formisano@policliniconapoli.it (T.F.); roberta.bottino@policliniconapoli.it (R.B.); saverio.delia@policliniconapoli.it (S.D.); rosa.franzese@policliniconapoli.it (R.F.); daniele.molinari@policliniconapoli.it (D.M.); andreina.carbone@policliniconapoli.it (A.C.); pasquale.castaldo@policliniconapoli.it (P.C.); massimiliano.orlandi@policliniconapoli.it (M.O.); simona.sperlongano@unicampania.it (S.S.); alberto.palladino@policliniconapoli.it (A.P.); consiglia.barbareschi@unicampania.it (C.B.); 2 Department of Advanced Medical and Surgical Sciences, University of Campania Luigi Vanvitelli, Piazza Luigi Miraglia, 2, 80138 Naples, Italy; 3 Department of Public Health, University of Naples “Federico II”, 80131 Naples, Italy; 4 Department of Translational Medical Sciences, Section of Cardiology, University of Campania Luigi Vanvitelli, 80131 Naples, Italy

**Keywords:** stroke, migraine, decompression sickness

## Abstract

Patent foramen ovale (PFO) is a condition present in 20–25% of the general population and can rarely be associated with clinical conditions such as stroke, decompression sickness, desaturation, and migraine with aura. Screening for PFO for primary prevention is not recommended. Several trials have been published demonstrating the efficacy of percutaneous PFO closure in patients under 60 years of age with embolic stroke of unknown etiology. Such evidence has not been achieved in other patient groups, such as those with migraine with aura or decompression sickness. There is also a subset of patients, such as pregnant women or patients undergoing laparoscopic non-cardiac surgery, in whom an increased risk of paradoxical embolism requires preoperative stratification. In all cases, it is necessary to characterize the PFO based on the size of the shunt and the anatomy (e.g., the presence of a large tunnel, prominent Eustachian valve, atrial septal aneurysm), all factors that can increase the risk of paradoxical embolism. This review summarizes the current view on diagnostic and therapeutic work-up of PFO in light of the most recent international guidelines.

## 1. Introduction

Patent foramen ovale (PFO) is a physiological communication between the right and left atrium necessary for the fetal circulation. At birth, this communication closes through an endothelization process within the first year of life. However, a persistent PFO is found in approximately 20–25% of the general population [1,2]. Data demonstrate substantial, modality-driven heterogeneity in reported prevalence among healthy control cohorts [3]: 24.2% in autopsy series, 23.7% at transesophageal echocardiography (TEE), 31.3% at transcranial Doppler study (TCD), 14.7% at transthoracic echocardiography (TTE). We performed a non-systematic review of the literature by applying the search strategy in different electronic databases (MEDLINE, EMBASE, Cochrane Register of Controlled Trials, and Web of Science). Original reports, meta-analyses, and review articles in peer-reviewed journals up to May 2026 regarding the epidemiology, the embryology, and, above all, the impact of PFO in various clinical scenarios were considered.

Regarding demographic distribution, PFO prevalence exhibits sex-equivalence, with no statistically significant variance between men and women [4,5]. In terms of chronological trends, while a PFO represents a permanent anatomical variant following a failure of physiological fusion during infancy, select post-mortem data indicate a marginal decline in both prevalence and probe-patent diameter during senescence [5]. This late-stage attrition is hypothesized to arise from continuous, delayed anatomical closure or age-related alterations in atrial tissue compliance. Consequently, the vast majority of affected individuals remain entirely asymptomatic throughout their lifespan, with the patency discovered merely as an incidental finding [6]. In the last three decades, more robust evidence has emerged on the pathogenic role of PFO in various clinical scenarios; therefore, the European Association for Percutaneous Cardiovascular Interventions [7], American Heart Association [8] and European Stroke Organization [9] have published updated guidelines to which we refer in our review.

The epidemiological landscape shifts dramatically when evaluating patients presenting with ESUS or cryptogenic stroke, revealing a distinct age-dependent distribution:

- The Young Stroke Cohort (≤60 years): In younger individuals presenting with cryptogenic events, PFO prevalence escalates sharply to 40–50%, with select registries reporting an incidence as high as 55% in patients under 55 years of age who lack traditional cardiovascular risk factors [10].
- The Older Stroke Cohort (>60 years): Although historically conceptualized as a pathology predominantly affecting younger populations, contemporary epidemiological data confirm that PFO prevalence remains elevated in older cryptogenic stroke cohorts as well. However, the etiological probability and the PFO-attributable fraction drop substantially in this demographic. This decline is driven by the confounding background noise of competing atherosclerotic and cardioembolic substrates—such as occult paroxysmal atrial fibrillation—which increase exponentially with age [11].

## 2. Embryology of PFO

Foramen ovale represents the persistence of a physiological interatrial communication that arises during normal cardiac embryogenesis [12]. The PFO differs from an interatrial septal (IAS) defect because it is not a lack of tissue but a deficit of obliteration of a virtual space between the two layers of septum primum and septum secundum. Formation of the interatrial septum is a complex, sequential process involving the coordinated development of the septum primum and septum secundum [12,13] starting in the fourth week of gestation. The septum primum arises from the posterior superior wall of the common atrium and grows towards the endocardial cushions, delimiting the ostium primum, which permits right-to-left shunting in early gestation. Prior to the closure of the ostium primum, programmed cell death within the superior portion of the septum primum generates the ostium secundum, thereby preserving interatrial flow [12]. Subsequently, the septum secundum forms to the right of the septum primum as a more robust, crescent-shaped structure, leaving a residual oblique channel, the foramen ovale [12]. In the fetal circulation, the foramen ovale allows oxygenated placental blood to stream from the right atrium to the left atrium, bypassing the high-resistance pulmonary circulation [12,14]. At birth, lung expansion and the consequent reduction in pulmonary vascular resistance lead to a rapid increase in pulmonary venous return and left atrial pressure, exceeding right atrial pressure [12]. This hemodynamic change results in functional apposition of the septum primum against the septum secundum, thereby closing the foramen ovale [12]. In the majority of individuals, this apposition is followed by anatomical fusion during early postnatal life [12].

## 3. When to Search for a PFO

According to recent expert consensus documents [7,9], the search for a PFO is recommended in the following conditions:

- Stroke of unknown origin.
- Decompression sickness.
- Desaturation syndrome.
- Refractory hemicrania with aura.

It is not recommended to search for a PFO in primary prevention.

### 3.1. Ischemic Stroke and PFO

PFO is present in 40–50% of young patients (age ≤ 60 years) with embolic stroke of undetermined source (ESUS). Ischemic stroke represents 88% of total strokes, and, among the ischemic strokes, 77% are non-lacunar, and 23% are lacunar. A lacunar stroke is a small stroke, with a radiologic area smaller than 1.5 cm, caused by small vessel disease. Among non-lacunar strokes, 45% are cryptogenic, 35% are cardioembolic, 17% are caused by large vessel (atherosclerosis or dissection) and 3% other rare causes (e.g., infectious). ESUS is a subcategory of cryptogenic stroke, which includes ESUS and non-ESUS stroke. ESUS is a non-lacunar stroke with a radiologic aspect of embolic stroke caused by an embolus of unknown origin [15,16]. A PFO-related stroke has the aspect of a non-lacunar stroke of embolic origin.

**Figure 1:**
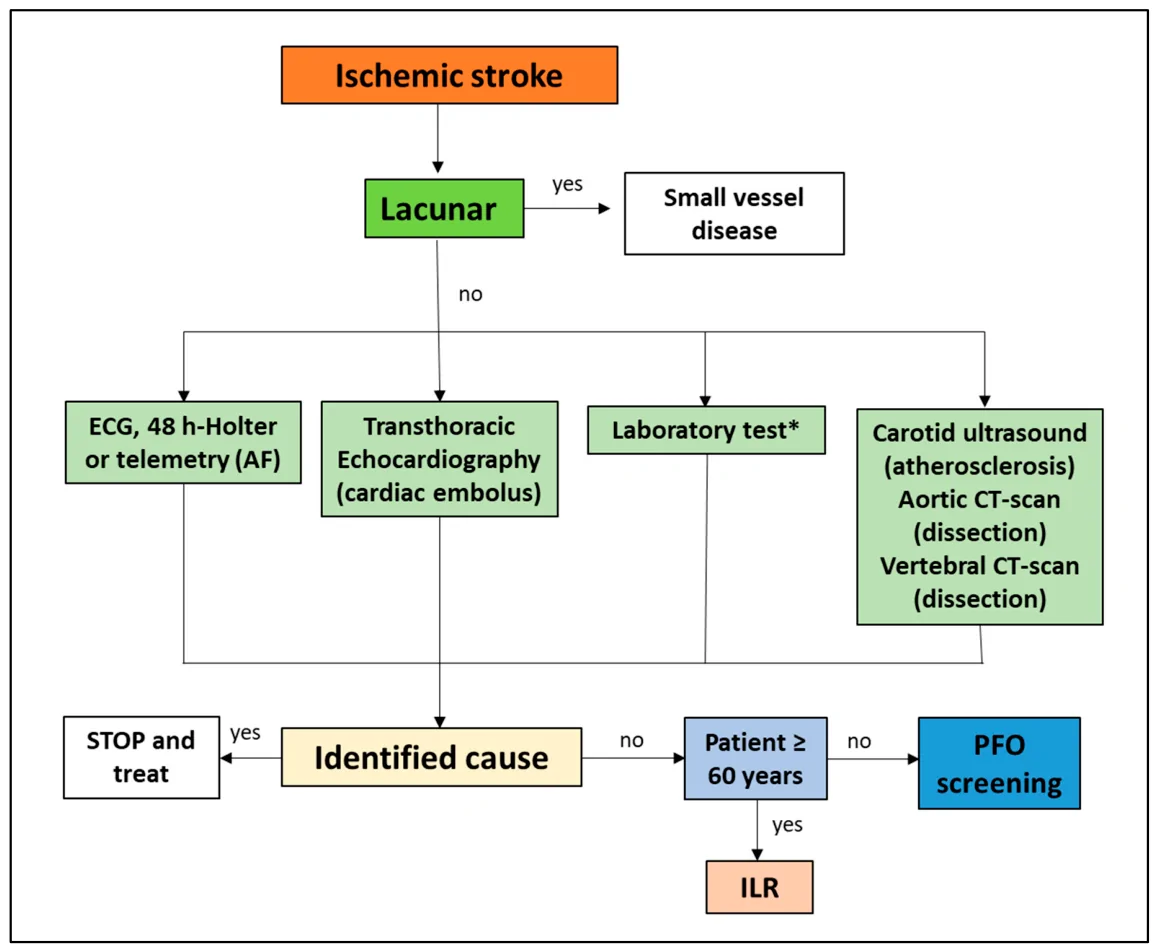
Diagnostic flow-chart of stroke. CT: computed tomography; ILR: implanted loop recorder. * blood sugar, cholesterol, red blood cells, platelets, CRP, thrombophilia tests.

In order to rapidly identify the cause of an ischemic stroke, the European Stroke Organization (ESO) [9] and American Heart/American Stroke Association (AHA/ASA) [16] recommend a first-level diagnostic work-up (Figure 1):

1. Radiologic test to confirm the diagnosis of stroke (brain CT, or if available, MRI).
2. Electrocardiogram and telemetry or 24 h Holter monitoring to exclude atrial fibrillation (AF).
3. Transthoracic echocardiography to exclude vegetations, left ventricular thrombi, and cardiac tumors.
4. Carotid ultrasound or computed tomography (for stroke of the anterior circulation) to find atherosclerotic plaques.
5. Aortic and vertebral computed tomography (to exclude dissection in strokes of posterior circulation).
6. Blood testing (glucose, lipid, antiphospholipid antibodies).
7. Venous Doppler ultrasound (to exclude deep vein thrombosis).

If no evident cause is found in the aforementioned panel exams, in younger patients (≤60 years) without risk factors for AF (e.g., heart failure, valvulopathies, cardiomyopathies) is mandatory to search for PFO, while in older patients (>60 years) or younger patients with AF risk factors, the loop recorder implantation is indicated [7,9,16]. Screening for PFO in the over-60 population is supported by a few randomized controlled trials [17,18] and should be limited to highly selected cases of ESUS where the cause remains unidentified even after loop recorder implantation.

### 3.2. Diagnosis of PFO

In most patients with PFO, there is no passage (shunt) of blood from the left to right atrium at rest, but in some conditions (such as the Valsalva maneuver), a transitory right-to-left shunt can occur, being the predisposing condition to paradoxical embolism. However, in the case of elevated left atrial pressure (for example in the presence of severe mitral stenosis) and concomitant PFO, a persistent left-to-right shunt can occur as a consequence of a high-pressure gradient. The PFO can also be associated with an interatrial septal aneurysm, defined by a maximum excursion of more than 10 mm of the IAS with respect to a basal plane passing through an ideal linear IAS.

Transthoracic echocardiography (TTE) is the first diagnostic approach for the diagnosis of PFO, with a variable sensitivity among studies, ranging from 46 to 91% and high specificity (99%) [19].

The subcostal view and the apical four-chamber view allow the visualization of the interatrial septum and, rarely, the passage of flow with color Doppler. The accuracy of TTE is higher when combined with the bubble test. TTE with bubble test should be performed at baseline and after Valsalva maneuver. The appearance of bubbles in the first three cycles after the opacification of the right atrium at the end of agitated saline bolus infusion is considered diagnostic for PFO. The diagnostic accuracy of TTE is limited by the maintenance of a good window during the Valsalva maneuver.

The TCD with bubble test has a sensitivity of 97% and specificity of 93% for diagnosing a right-to-left shunt [6] when compared to transesophageal echocardiography (TEE) and a sensitivity of 98% and accuracy of 94% when compared to cardiac catheterization.

The passage of bubbles through a PFO generates microembolic signals (MES) detected during the registration of power Doppler signals of the middle cerebral artery. A minimum of 1–3 signals (MES) registered in the first 3–5 cardiac cycles at the end of bolus infusion are considered sufficient to diagnose a right-to-left (R-L) shunt. When the signals are registered after the first five cycles, it is suggestive of an extra-cardiac shunt (for example, an artero-venous pulmonary fistula) and a CT lung scan is necessary to confirm [20].

Usually, TCD and TTE with bubble test are performed in one session. If TCD or/and TTE are positive for the presence of R-L shunt, transesophageal echocardiography (TEE) is mandatory to confirm the diagnosis.

The sensitivity of TEE to detect PFO is 89%, lower than TCD, probably due to the difficulty of performing an adequate Valsalva’s maneuver during intubation. However, TEE has higher diagnostic accuracy than TCD and TTE to define the anatomy, the presence of a Chiari’s network, a redundant Eustachian valve, and an interatrial septal defect. In the presence of an extracardiac shunt, such as an arteriovenous pulmonary fistula, opacification of one pulmonary vein can be observed after the administration of agitated saline solution [21]. Furthermore, it is an essential preoperative tool to evaluate the suitability of transcatheter PFO closure, which requires the presence of adequate anatomic rims to anchor the device.

**Figure 2:**
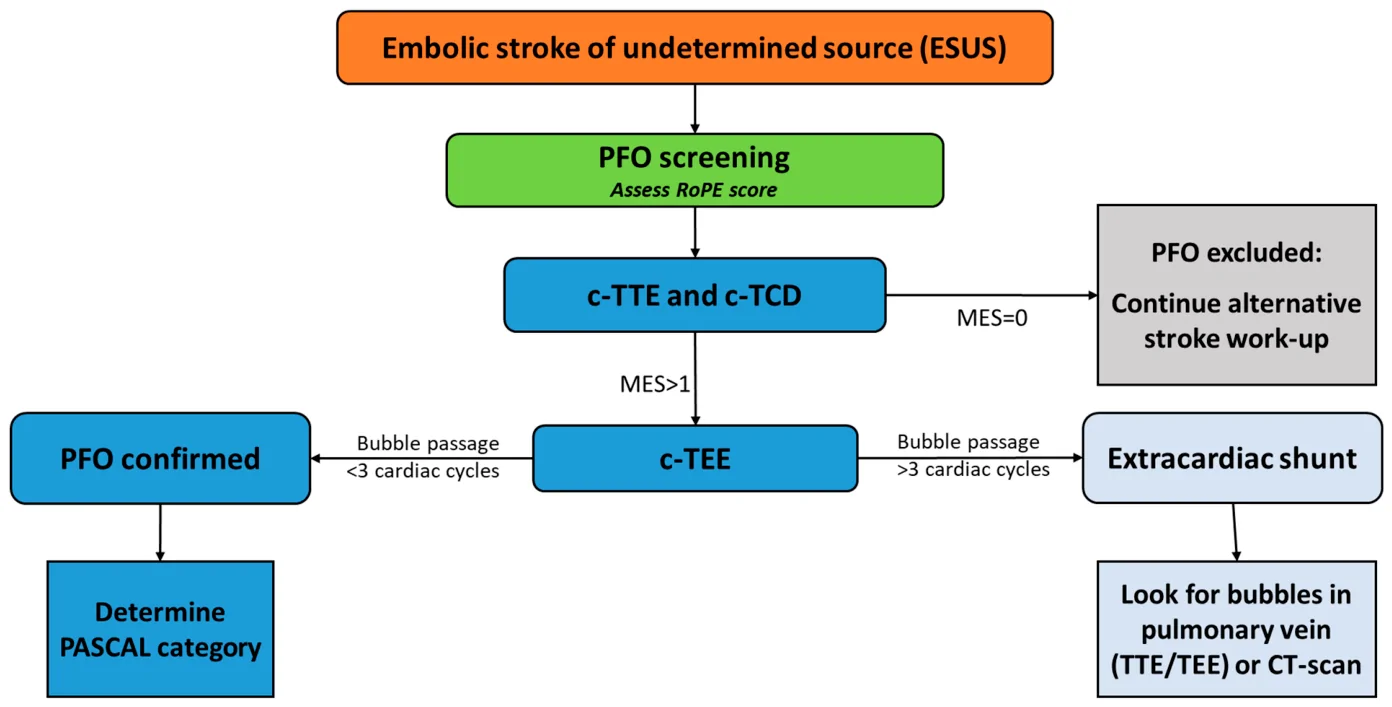
Diagnostic work-up in embolic stroke of undetermined source. RoPE: risk of paradoxical embolism; PFO: patent foramen ovale; TTE: transthoracic echocardiography; TCD: contrast-transcranial Doppler; TEE: contrast-transesophageal echocardiography; MES: microembolic signal; PASCAL: patent foramen ovale-associated stroke causal likelihood; c: contrast (bubble test: 1 mL air in 9 mL saline agitated solution); CT: computed tomography.

According to current evidence, there is no gold standard for the diagnosis of PFO with an R-L shunt, but the combination of TTE, TCD and TEE is necessary to achieve an accurate diagnosis (Figure 2).

#### Transcranial Doppler Ultrasound: The Correct Procedure

The transcranial Doppler is performed by using a 2-Mhz sectorial probe positioned in the temporal bone region to sample the flow of the middle cerebral artery by power Doppler function. A sample volume of 8 mm in length and a low gain were used, and the patient was placed in a supine position. An 18-gauge catheter with a three-way stopcock was positioned in the right antecubital vein to administer agitated saline solution prepared by vigorously mixing 9 cc of saline and 1 cc of air in a three-way catheter. One cc of blood can alternatively be mixed with saline, but we do not recommend it due to a greater biological risk for the operator. When the prepared solution is administered, high-intensity transient signals (HITS), defined as visible and audible short-duration signals, are recorded by an ultrasound probe. In case of little or no detection of HITS under basal conditions, the examination is repeated using a 10 s Valsalva maneuver (VM) started 5 s after the injection on the examiner’s command. Before the procedure, the patients must be trained to perform the VM correctly and the strength of the VM controlled by a peak flow velocity decrease of at least 25% on the Power Doppler signal [6,20]. In case of no detection of HITS, the test with VM should be repeated twice. The test is considered positive when at least 1 HITS is recorded within 40 s of the injection; however, in the presence of shunt, the MES often appear in the first five cycles. The magnitude of RLS is determined by counting the number of HITS in the MCA in the first 40 s after saline administration [21]. False negatives may be related to a poor acoustic window or an inadequate Valsalva maneuver. When the temporal window is not suitable, the TCD can be performed by using occipital or orbital positions to sample the flow of the anterior cerebral and posterior cerebral artery. To increase the accuracy, some authors use the upright sitting position. There are two grading system to quantify the R-L shunt: the 4 level International Consensus Criteria scale [22] (ICC; 0 = no shunt; grade I = 1–10 MES, grade II = > 10 MES but without curtain or shower effect; grade III = curtain or shower effect) and 6-level Spencer Logarithmic Scale [23] (grade 0 = absence of MES; grade I = 1–10 signals; grade II = 11–30 signals; grade III = 31–100 signals; grade IV = 101–300 signals; grade V > 300 signals). The most widely used is the ICC scale because a cut-off ≥ 10 bubbles at TCD or ≥20 bubbles at TTE or TEE is considered sufficient to consider PFO as a possible cause of ischemic stroke [7,9].

### 3.3. When to Close PFO in ESUS

Given the high prevalence of PFO in the general population, it is necessary to establish a causative role of PFO in patients with ESUS. The results of a meta-analysis of six RCTs have demonstrated that PFO closure reduces the risk of recurrence of stroke/TIA compared to medical treatment [7]. After the closure, the risk of recurrence is lower in the absence of cardiovascular risk factors and in younger patients. In order to help the physician to establish a causative role of PFO in ESUS, the RoPE (risk of paradoxical embolism) score and the PASCAL (PFO-associated stroke causal likelihood) system classification have been introduced (Table 1).

The ROPE score assigns a score from 0 to 10 to the patient, where 10 is the maximum and zero is the minimum probability that the stroke is PFO-related (Table 2).

The ROPE score considers the age and the absence of cardiovascular risk factors, and a cut-off ≥ 7 is considered significant for the causality of PFO. Another classification system has been proposed (Pristipino, Caso) to define the probability of a causal link between PFO and ESUS, the PASCAL system, which combines the likelihood of the RoPE score with anatomic risk factors of PFO (Table 3).

In fact, the probability that the PFO was the cause of ESUS is higher when it is associated with large shunts (more than 10 bubbles at TCD to more than 20 bubbles at TTE or TEE) or with the aneurysm of the interatrial septum (ASA). This score system came out of a meta-analysis demonstrating that patients with large shunts and concomitant ASA have a greater benefit than patients with small shunts and ASA or large shunts without ASA [24] after PFO closure.

**Figure 3:**
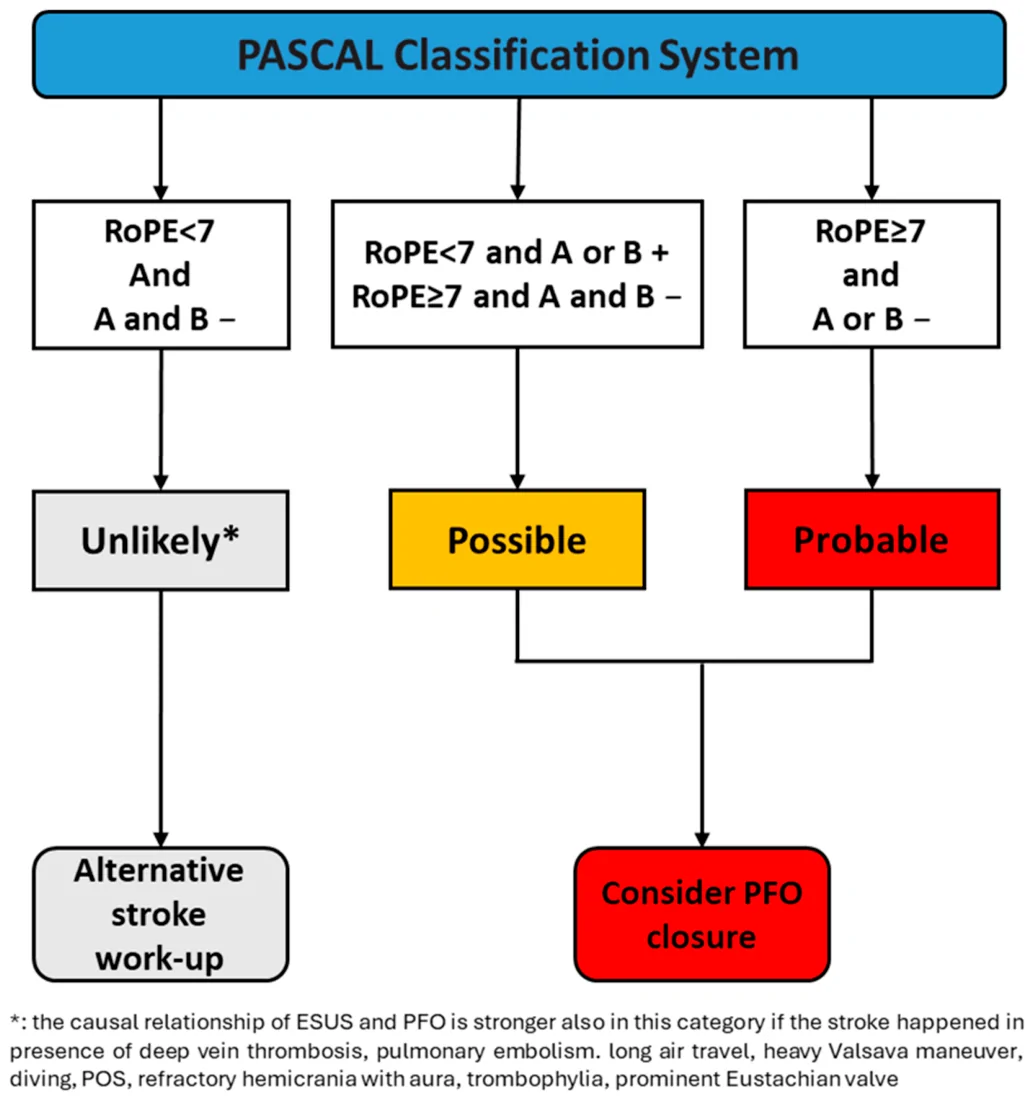
Decisional algorithm in patients with ESUS and PFO. PASCAL: patent foramen ovale-associated stroke causal likelihood; RoPE: risk of paradoxical embolism; ESUS: embolic stroke of undetermined source; PFO: patent foramen ovale. The sign “+” means presence, the sign “−” means absence.

Both the ROPE score and PASCAL system have to be considered in the causal relationship between ESUS and PFO. In the presence of both ROPE score ≥ 7 and a “high-risk” feature, the causal relationship is “probable”, while in the presence of only one among the ROPE score ≥ 7 and a “high-risk feature”, the relationship is considered “possible”. In cases of “probable” or “possible”, it is recommended to close the PFO because the odds ratio of risk of recurrence is lower in the treated group. If the ROPE score is less than seven and the PASCAL score is negative, the causal relationship is considered “unlikely,” and PFO closure is not recommended [7,9]. However, if the patient is in the “unlikely” category, there are some conditions associated with ESUS, such as concomitant deep vein thrombosis, decompression disease, intense Valsalva maneuver, and long air travel, which increase the causal relationship and justify the indication for PFO closure [7,9] even in this category. The use of the PASCAL system is indicated in patients aged between 18 and 60 years (Figure 3).

Indeed, the PASCAL system—extrapolated from meta-analyses of patients undergoing PFO closure—is not validated in patients over 60 and should not be used in this category of patients. In patients older than 60 years, it is mandatory to search for silent atrial fibrillation by monitoring with a loop recorder (LR). The European Society recommended the loop recorder also in patients younger than 60 years with AF risk factors (for example, heart failure with reduced or preserved ejection fraction, severe or long-term arterial hypertension, valvular disease, obesity, thyroid disorders).

### 3.4. Decompression Sickness and PFO

Decompression sickness (DS) is due to rapid atmospheric pressure reduction in divers or flyers, presenting a variable clinical scenario with mild to more severe symptoms. DS incidence in sport diving is three cases per 10,000 dives, higher among commercial divers ranging from 1.5 to 10 per 10,000 dives [25]. The incidence is lower in pilots, reported as 0.23% in high-altitude flight among U2 aircraft pilots [7].

There are two known mechanisms of DS: the air embolization causing ischemic symptoms and the endothelial rupture due to accumulation of bubbles in the microcirculation (autochthonous damage) [26]. Ascending from a dive or rapidly reaching high altitudes causes a rapid atmospheric pressure reduction and, therefore, an expansion of alveolar gas and endothelial damage (barotrauma), with air shifting into the pulmonary circulation causing pulmonary edema and cerebral ischemia [26]. The diffusion of air emboli can also affect other organs, such as the heart, resulting in ischemic symptoms. Another mechanism involves the production of local emboli of nitrogen and oxygen, causing endothelial damage, especially of the vestibulocochlear system, the skin, the joints, the spinal cord and central nervous system. The symptoms are variable and can include skin reactions, headache, dizziness, arthralgia, paraplegia, hyposthenia and various stroke-like symptoms. In the DS, the presence of a PFO does not play a central role, but it can predispose to arterial embolization through the passage of gas emboli from the venous to arterial circulation. In small retrospective and case–control studies, a higher prevalence of PFO has been observed in divers with cerebral DS and vestibulocochlear syndrome compared to divers without DS or with non-cerebral DS [27,28,29]. The results of a meta-analysis of four studies reported an increased incidence of PFO with R-L shunt in patients with DS, with an odds ratio of 5.63 (95% CI: 3.14–10.09) [2].

The diagnosis of DS is clinical and based on risk factors (prolonged diving or flying with rapid ascent). However, in the immediately following hours, circulating microbubbles can be observed via echocardiography.

#### When to Search and When to Close PFO in Decompression Sickness

Current guidelines [2] recommend as first approach to identify and correct predisposing factors to DS: prolonged immersion, rapid ascent, alcohol consumption, obesity, smoking, dehydration. In the absence of these risk factors or if a second episode occurs after correcting the risk factors, experts recommend searching for PFO and performing pulmonary tests (such as CT lung scans and spirometry) to detect any pre-existing lung disease. If a PFO is diagnosed, the decision must be taken on a case-by-case basis among [2] the following options:

- Stop the activity.
- Modify the diving or flying profile.
- Close the PFO.

However, professional divers often exhibit a higher reluctance to modify the diving protocol, preferring to close the PFO. An interesting trial on 829 asymptomatic divers [30] was conducted to search for PFO using TCD and TEE. Those with a severe R-to-L shunt (with curtain or shower effect) were randomized to percutaneous closure or a conservative strategy. In the conservative group. After a long follow-up (6.5 ± 3.5 years), a higher risk of DS was observed (hazard ratio: 26.170; 95% CI: 5.797–118.160; *p* < 0.0001).

To date, except for one study [30], the available data are mostly derived from observational retrospective studies [31] or small trials [32], with only one multicenter trial published, and more evidence deriving from randomized controlled trials is necessary to provide more solid recommendations. Currently, it is not recommended to search for PFO in divers or flyers for primary prevention, considering the high prevalence of PFO in the general population and the low risk of DS in these categories. There are issues associated with PFO closure in divers: first, a pathogenic role of PFO in DS has been demonstrated; thus, the closure must be effective—leaving no residual shunt—to successfully reduce risk. Finally, it should be considered that the procedure does not eliminate the residual risk of DS linked to other mechanisms, such as barotrauma or local tissue damage. Therefore, in cases of DS, it is advisable to determine the treatment strategy following a multidisciplinary assessment involving a hyperbaric medicine specialist, a cardiologist, and a neurologist.

### 3.5. Desaturation Syndrome and PFO

The PFO-related desaturation syndrome, diagnosed when SpO2 at rest is < 90% or a reduction > 8% during effort [2], is a rare condition characterized by desaturation, especially in the standing position or dyspnea during effort due to high volume R-L shunt. The predisposing factors for this condition are:

- High right atrial pressure caused by atrial arrhythmia, tricuspid stenosis or regurgitation, pulmonary hypertension, chronic obstructive pulmonary disease.
- Favorable anatomy due to large Eustachian valve that directs the flow preferentially towards the septum.

The PFO-related desaturation syndrome could occasionally be diagnosed [33] or in a context of desaturation sine causa.

Platypnea-orthodeoxia syndrome is a subcategory of PFO desaturation syndrome characterized by desaturation and dyspnea in the upright position which resolves in the supine position. It is a rare condition, and a small observational study demonstrates [34] the efficacy of percutaneous closure for both saturation and symptoms after PFO closure.

In a patient with desaturation syndrome, more frequent causes must first be excluded (lung disease, pulmonary embolism, pulmonary hypertension, congenital heart disease) before considering PFO as the cause of desaturation.

In this situation, the diagnosis of PFO follows the aforementioned diagnostic panel (Figure 2), but if the TCD results are negative in the presence of high suspicion, the test should be performed in an upright position and/or by using the femoral vein to inject saline solution, as the blood flow of the inferior vena cava preferentially goes towards the PFO.

There are no RCTs to test the efficacy of closing PFO in these rare conditions, but a small meta-analysis(2) demonstrates an increase in exercise desaturation of 9.8% (95% CI: 7.1–12.5%) with a severe heterogeneity among studies (I2: 79%) and in POS of 9.6% (95% CI: 5.7–13.5%) also with a severe heterogeneity among studies (I2: 82%).

In patients with obstructive sleep apnea, the prevalence of PFO with large to severe R-to-L shunt is higher than in the general population (69% vs. 17%) [35], probably due to repeated Valsalva and Muller maneuvers favoring the R-to-L shunt. However, small observational studies have shown controversial results [35,36] regarding the beneficial effect of transcatheter PFO closure on the OSAS disease burden, leading to the conclusion that the PFO does not always play a central role in the pathogenesis of hypoxia in OSAS patients. To date, no recommendations exist for this subset of patients, and the decision to close PFO should be made only in specific cases in which a significant desaturation is registered during the TCD.

### 3.6. Migraine and PFO

Migraine is a disabling neurological disorder defined by the International Classification of Headache Disorders (ICHD-3) as a recurrent headache manifesting in attacks lasting from 4 to 72 h [37]. Characteristically unilateral, pulsating, and of moderate-to-severe intensity, it is typically aggravated by routine physical activity and associated with nausea, photophobia, or phonophobia [37]. Migraine can manifest with or without aura, the latter defined as unilateral, fully reversible visual, sensory, or central nervous system symptoms that develop gradually [37].

An established epidemiological link exists between migraine with aura (MwA) and patent foramen ovale (PFO). Patients suffering from MwA exhibit a significantly higher PFO prevalence (41% to 48%) than the general population, suggesting the hypothesis of a pathogenic link between interatrial shunting and migraine symptoms [38,39]. Several pathophysiological mechanisms have been proposed to explain this association. One prominent humoral hypothesis suggests that vasoactive substances, such as serotonin, or microemboli bypass the pulmonary filter via a right-to-left shunt (RLS) to enter the systemic circulation directly and without their usual pulmonary metabolic degradation, allowing these agents to reach the cerebral vasculature and trigger trigeminal nerve irritation [40]. Additionally, transient paradoxical embolism may induce focal cortical hypoxia or provoke serotonin-mediated platelet activation. Such microthrombi could lower the threshold for cortical spreading depression (CSD), precipitating migraine attacks [40]. This clinical relationship is heavily influenced by the anatomical and functional characteristics of the PFO. Evidence suggests a direct correlation between RLS magnitude, concomitant atrial septal aneurysm (ASA) and migraine history and number of attacks [41,42,43], even though PFO anatomical features and size do not seem to correlate with clinical symptoms [41,42,43].

Interestingly, in migraineurs with PFO, the incidence of permanent PFO is higher than that of latent PFO (RLS occurring only after Valsalva maneuver) [44]. Despite these insights, the precise mechanism remains incomplete, and the overall link between PFO, migraine and aura carries inherent uncertainties.

Since the early 2000s, observational data demonstrated migraine reduction following percutaneous PFO closure, providing clinical support for this premise [45,46]. However, early investigations were hindered by small cohorts, retrospective designs, or selection bias—particularly the inclusion of patients with prior cerebrovascular events whose distinct natural history confounds outcomes [45,46,47].

To address these limitations, the MIST trial (2008) was launched as the first prospective, multicenter, randomized, sham-controlled study evaluating whether PFO closure could reduce or eliminate migraine attacks [48]. The study enrolled 147 patients (74 closure, 73 sham-control) with frequent, refractory MwA. The STARFlex device was used in PFO closures. Despite its rigorous design, the trial failed its primary endpoint of total migraine cessation, with only three patients (~4%) in each arm achieving complete resolution. Critical limitations were subsequently identified: the trial was underpowered due to high screen-failure rates and a short follow-up, and the STARFlex device carried high rates of residual shunting and atrial arrhythmias, offsetting potential benefits. Lastly, the study failed to stratify patients by shunt magnitude, pooling small or transient shunts with large, continuous shunts more likely to benefit from intervention. These shortcomings emphasized the need for refined patient selection and alternative device technology in subsequent research [49].

Following MIST, the PRIMA trial (2016) refined the clinical approach by exclusively enrolling 107 participants with chronic MwA unresponsive to at least two prophylactic medications, comparing the Amplatzer PFO Occluder against medical management [50]. Like its predecessor, PRIMA missed its primary endpoint of reducing mean monthly migraine days. However, a significant reduction in the frequency of migraine with aura attacks was observed in the closure group, suggesting that PFO-mediated shunting might be specifically linked to the aura phase rather than the headache itself. Despite these insights, the study was terminated prematurely due to slow recruitment, limiting its statistical power. Concurrently, the PREMIUM trial (2017) investigated a larger cohort of 230 patients randomized to either the Amplatzer PFO Occluder or a sham-control regimen [51]. While PREMIUM also failed its primary endpoint (a 50% reduction in total attacks), complete migraine cessation was achieved in 10% of the closure group versus 1% of controls. Furthermore, post hoc analyses indicated that patients with a large RLS experienced a more substantial clinical benefit, reinforcing the hypothesis that PFO closure offers therapeutic value to a specific subset of patients characterized by large shunts and prominent aura [51].

Following PREMIUM, the clinical landscape shifted from initiating independent randomized controlled trials (RCTs) to synthesizing existing datasets and refining patient selection paradigms. A pivotal patient-level pooled analysis of the PRIMA and PREMIUM trials demonstrated that while individual trials failed primary endpoints due to pronounced placebo effects, the combined dataset revealed a statistically significant reduction in monthly migraine days, lower overall attack frequencies, and a 9% rate of complete cessation compared to 1% in the medical cohort [52]. Recognizing that previous RCTs were limited by incidental rather than causal PFOs, contemporary research emphasizes stringent physiologic screening. This is exemplified by the novel ongoing GORE RELIEF study (NCT04100135), which utilizes prospective responsiveness to P2Y12 platelet inhibitors as an inclusion criterion to isolate microemboli-driven migraine phenotypes before randomization. Until data from such biomarker-targeted RCTs are fully integrated into professional guidelines, PFO closure remains restricted to highly selected cases of MwA not responsive to at least two-line pharmacological treatments as compassionate use and is not recommended as a routine, first-line intervention for isolated migraine.

PFO closure remains restricted to highly selected cases of MwA not responsive to at least two-line pharmacological treatments as compassionate use and is not recommended as a routine, first-line intervention for isolated migraine. Outside of selected cases, there is no evidence supporting routine closure of PFO in patients with migraine with aura. This aspect may be linked to the multifactorial etiology of this disease, which has not yet been fully elucidated and thus the lack of robust data coming from RCTs.

This conservative position is explicitly upheld by the European Association of Percutaneous Cardiovascular Interventions (EAPCI) [2], which recommends against routine device occlusion due to the lack of robust primary endpoint efficacy in randomized trials. Furthermore, the National Institute for Health and Care Excellence (NICE) classifies the evidence for migraine prophylaxis via PFO closure as inadequate in both quality and quantity, restricting the procedure to clinical trials or specialized governance frameworks [51,53]. Consequently, patient selection for device implantation remains governed by a stringent multidisciplinary framework centered first on the secondary prevention of recurrent paradoxical embolism.

## 4. Treatment of PFO

The therapeutic management of PFO has significantly evolved over the last decade following randomized trials, updated international guidelines, and emerging real-world evidence. In the past, there was no superiority of transcatheter closure of PFO over antithrombotic therapy, and the choice of treatment depended on practitioner and patient preference.

To date, patient selection remains essential because PFO is present in approximately 25% of the general population and is often incidental rather than pathogenic [54].

Patients most likely to benefit from closure include younger individuals with cryptogenic embolic stroke, cortical infarcts, absence of major vascular risk factors, large shunts, or associated atrial septal aneurysm [54,55].

The risk of paradoxical embolism (RoPE) score and the more recent PASCAL classification system are useful to select patient candidates for closure [7,9].

Conversely, closure is generally discouraged in patients with lacunar stroke, advanced atherosclerotic disease, atrial fibrillation, or alternative cardioembolic sources. Multidisciplinary evaluation involving neurologists and cardiologists is strongly recommended.

To date, after having established the causal role of PFO with the clinical events (ESUS, desaturation syndrome, decompression sickness, migraine), recent RCTs demonstrated a superiority of transcatheter device closure over medical treatment, even if the best evidence regards above all patients with ESUS [55,56,57,58].

### 4.1. Transcatheter Device Closure

Randomized controlled trials established transcatheter PFO closure as an effective strategy for secondary prevention of ischemic stroke in selected patients [55,56,57]. Early studies such as CLOSURE I and the PC Trial initially failed to show clear superiority over medical therapy [56,57]. However, subsequent analyses and longer follow-up demonstrated significant benefits. The RESPECT extended follow-up trial demonstrated a reduction in recurrent ischemic stroke after closure [59]. Similar benefits were confirmed in the REDUCE and CLOSE trials, especially among patients with large right-to-left shunts and atrial septal aneurysm (ASA) [10].

The DEFENSE-PFO trial [11] further supported closure in patients with high-risk anatomical features. Recent meta-analyses published in 2025 confirmed a substantial reduction in recurrent stroke risk with PFO closure compared with medical therapy alone [60].

The 2024 European Stroke Organisation (ESO) guidelines [9] recommend percutaneous PFO closure in patients aged 18–60 years with probable PFO-associated stroke after exclusion of alternative etiologies. Emerging evidence also suggests possible benefits in carefully selected patients older than 60 years [61,62].

#### 4.1.1. Suture-Mediated PFO Closure

In recent years, increasing attention has focused on suture-mediated (“deviceless”) PFO closure techniques, particularly with the NobleStitch EL system [63]. Unlike traditional occluder devices, this approach approximates the septum primum and septum secundum using polypropylene sutures without leaving a permanent metallic implant.

Potential advantages of suture-mediated closure include absence of intracardiac prosthetic material, preservation of interatrial septal anatomy, reduced risk of nickel hypersensitivity, and potentially lower rates of atrial arrhythmias [64].

The NobleStitch Italian Registry demonstrated feasibility and procedural safety in selected anatomies [63]. More recent long-term observational data published in 2025 reported low recurrence rates of stroke or transient ischemic attack and a very low incidence of atrial fibrillation [64,65].

However, residual shunt appears more frequently after suture-mediated closure compared with conventional device closure, particularly in patients with complex septal anatomy or large PFO tunnels [66,67]. Recent studies introduced the LASSO score to identify anatomies most suitable for suture-mediated closure [65].

Although randomized comparisons with conventional occluder devices are still lacking, suture-mediated closure represents one of the most promising technological developments in structural heart interventions [65].

#### 4.1.2. Procedural Aspects and Long-Term Outcomes

Percutaneous PFO closure is usually performed through femoral venous access under fluoroscopic and echocardiographic guidance [7]. Contemporary devices include the Amplatzer PFO Occluder and the Gore Cardioform Septal Occluder.

Technical success rates exceed 95%, with low rates of major complications [7]. The most common adverse event after closure is atrial fibrillation, usually transient and peri-procedural [61,68]. Recent real-world studies published between 2024 and 2026 demonstrated that long-term ischemic stroke recurrence after closure remains low and comparable to randomized clinical trials [61,69]. However, patients undergoing closure still demonstrate higher stroke risk compared with the general population due to their baseline cerebrovascular risk profile [69].

Current post-procedural management generally includes dual antiplatelet therapy for 3–6 months followed by long-term single antiplatelet therapy [9].

#### 4.1.3. Complications of PFO Transcatheter Closure

While percutaneous closure of a patent foramen ovale (PFO) effectively reduces the risk of recurrent embolic stroke of undetermined source (ESUS), inherent procedural risks still demand targeted postoperative surveillance. The most frequent complications are new-onset atrial arrhythmias, principally atrial fibrillation (AF) and atrial flutter, followed by access-site vascular complications, and rare but severe mechanical events such as cardiac tamponade and device thrombosis.

Atrial Fibrillation: The true incidence of AF and flutter generally ranges between 2.8% and 5% [70]. A recent meta-analysis [71] confirmed that percutaneous PFO closure significantly increases the arrhythmic risk compared to medical therapy alone (RR: 2.27, 95% CI: 1.29–4.01, *p* = 0.009). Notably, the temporal distribution of these events is heavily clustered within the first month post-procedure. Beyond this timeframe, the difference between the two groups disappears (RR: 0.60, 95% CI: 0.02–19.88, *p* = 0.60), suggesting the involvement of transient triggers rather than permanent structural remodeling.

To date, the exact mechanisms underlying these post-procedural arrhythmias remain poorly understood. Unlike atrial septal defects (ASD), PFO carries no chronic volume overload or baseline structural alterations; thus, early arrhythmic onset is thought to arise from three main factors:

- Mechanical irritation and acute tissue stretch induced by device deployment, which generate local inflammation, scarring, and de novo macrore-entrant circuits [72].
- Transient alterations in left atrial biomechanics (specifically affecting left atrial longitudinal, reservoir, and contractile strain), which are established predictors of AF [73], and typically resolve completely within 6 to 12 months [74,75].
- An inflammatory response driven by nickel release into the bloodstream from the nitinol (nickel–titanium alloy) framework of the device prior to complete endothelization (1–3 months) [76]. The peak of nickel release occurs around the 14th post-procedural day, temporally coinciding with the window of maximum incidence of supraventricular tachycardias and its subsequent resolution within 3 months [77,78].

Despite these acute alterations, PFO closure does not increase the long-term risk of AF; 5-year data from a nationwide cohort demonstrated no significant differences in AF incidence compared to untreated PFO controls [79].

Regarding preventive strategies, a standardized approach is currently lacking. The recent randomized AFLOAT trial [80] demonstrated the inefficacy of flecainide in preventing post-closure arrhythmias (26.8% in the active group vs. 25.4% in the control group, *p* = 0.83).

Presently, no official, guideline-validated algorithm exists for the diagnosis of AF following PFO closure. Nevertheless, intensive surveillance is warranted within the first 45 days post-procedure, as 86.8% of arrhythmias—driven by transient inflammatory and procedural factors—occur during the first month, peaking at day 14 [80]. Conversely, AF onset beyond 45 days reflects the patient’s intrinsic vascular risk or pre-existing abnormalities(13). Accordingly, the 2024 European Stroke Organisation (ESO) guidelines do not support systematic implantable loop recorder (ILR) screening for all postoperative patients. Instead, ILR deployment should be reserved for specific scenarios, such as patients older than 60 years, recurrent cerebrovascular events post-closure despite unrevealing short-term monitoring, or the completion of ongoing monitoring for devices implanted prior to the procedure [9].

The detection of AF after PFO closure demands a personalized and comprehensive therapeutic strategy. Ideally, management should follow the simplified AF-CARE pathway promoted by recent guidelines [81]. However, the most complex clinical challenge lies in the prevention of thromboembolic risk and the selection of the optimal antithrombotic regimen (A pillar of AF-CARE). In this setting, the uncritical application of the CHA_2_DS_2_-VA score creates a clinical paradox: a history of prior stroke automatically assigns a score of two, formally mandating oral anticoagulation in patients who are otherwise young and healthy.

Nonetheless, experts agree that the actual necessity of oral anticoagulation therapy remains highly debated for three primary reasons:

1. The transient nature of post-PFO AF, which is frequently destined to resolve spontaneously within the first 45 days.
2. The risk of overtreatment, as management during the initial weeks must account for the concomitant antiplatelet therapy required for device endothelization; adding an oral anticoagulant would significantly expose the patient to an increased risk of bleeding.
3. The inadequacy of the CHA_2_DS_2_-VA score, which was validated in different patient populations and could potentially be replaced by a modified version that excludes prior stroke.

Consequently, real-world management remains heterogeneous and tailored case by case. Current paradigms propose distinguishing whether the AF is secondary (device-related, where anticoagulation should be avoided or administered only for brief periods) or primary (linked to a pre-existing substrate). The decision to initiate anticoagulation must rely on an integrated evaluation balancing the CHA_2_DS_2_-VA score, individual bleeding risk, and actual arrhythmia burden.

Finally, following the AF-CARE principle of dynamic reassessment (Evaluation), longitudinal follow-up is mandatory. If monitoring reveals persistent or late-onset AF (beyond 45 days post-intervention), the arrhythmia likely reflects the patient’s native substrate rather than a procedural insult. Consequently, its recurrence mandates a transition to full-dose, long-term anticoagulation, regardless of the closure device.

Other Complications: Other complications associated with percutaneous PFO closure exhibit a low overall incidence but include time-dependent and potentially life-threatening events.

Regarding vascular access-site complications, the utilization of medium-to-large-caliber femoral venous sheaths (8–12 Fr) exposes patients to the risk of hematomas, pseudoaneurysms, and arteriovenous fistulas. The in-hospital adverse event rate is reported at 7%, rising to 10.9% in individuals older than 60 years [82]. These vascular issues necessitate a meticulous puncture technique and rigorous post-procedural monitoring of vital signs and the access site to detect early signs of hypotension or a drop in hemoglobin levels.

Turning to intra-procedural mechanical complications driven by left atrial manipulation of guidewires and delivery systems, the most formidable are pericardial tamponade (0.17%) and the perforation of thin-walled structures (0.06%). Additionally, late device erosion represents a rare but noteworthy long-term risk, estimated at 0.018% [9].

## 5. Role of PFO in Special Settings: Pregnancy, Neurosurgery and Other Non-Cardiac Surgery

The epidemiological association between PFO and systemic embolization is well established [2,83]. However, specific clinical and surgical environments act as potent hemodynamic amplifiers, transforming an anatomically silent PFO into a high-risk conduit for systemic thrombotic or gaseous emboli by acutely reversing the interatrial pressure gradient (right atrial pressure [RAP] > left atrial pressure [LAP]).

### 5.1. Pregnancy

The gestational period and the peripartum phase introduce a complex matrix of biochemical and hemodynamic alterations that fundamentally disrupt PFO equilibrium:

- Hypercoagulable State: Pregnancy induces a profound systemic hypercoagulability via the upregulation of factors VII, VIII, X, and fibrinogen, alongside the downregulation of protein S and the inhibition of fibrinolysis via placental plasminogen activator inhibitor-1 (PAI-1) [84].
- Mechanical and Geometric Remodeling: By the third trimester, plasma volume expands by up to 50%. Mechanical compression of the inferior vena cava (IVC) by the gravid uterus leads to marked venous stasis in the pelvis and lower extremities, increasing the incidence of deep vein thrombosis (DVT). Geometrically, the displacement of the IVC axis redirects the venous bloodstream directly toward the fossa ovalis, enhancing blood flow and facilitating PFO opening even at rest [85].
- The Expulsive Phase Interatrial Shift: During the expulsive phase, repetitive and prolonged maternal Valsalva maneuvers drastically increase intrathoracic pressure, transiently reducing venous return. During the subsequent release phase, a massive surge of blood enters the right atrium, causing an acute spike in RAP that exceeds LAP. This sudden inversion of the interatrial pressure gradient forces open the compliant PFO flap, permitting pelvic or lower-limb microthrombi to pass into the systemic arterial circulation, culminating in cryptogenic ischemic stroke or peripheral embolic events [85,86].

Current European Society of Cardiology (ESC) and Italian Cardiology guidelines [86,87] emphasize that pregnant patients with a known high-risk PFO anatomy (e.g., associated with an atrial septal aneurysm [ASA] showing a mobile excursion > 10 mm (or a massive baseline shunt) or a history of cryptogenic stroke must be managed by a multidisciplinary Pregnancy Heart Team to individualize antithrombotic regimens (low-molecular-weight heparin [LMWH] or low-dose aspirin).

The presence of PFO is not a contraindication to vaginal delivery, although repeated Valsalva maneuvers during vaginal delivery may increase the R-to-L shunt; cesarean delivery should not be preferred, as it is burdened by a 4-fold increase in thromboembolic risk. Spontaneous or assisted vaginal delivery remains the gold standard [86]. To blunt the hemodynamic dangers of expulsive efforts, guidelines mandate the early initiation of effective lumbar epidural analgesia, which suppresses maternal labor pain, attenuates hyper-adrenergic blood pressure spikes, and crucially abolishes the involuntary urge to push, thereby minimizing the intensity and frequency of aggressive Valsalva maneuvers [86,88].

Some case reports have been published on patients with amniotic fluid embolism and PFO-related stroke during cesarean delivery [89,90]. To date, in pregnant women with asymptomatic PFO, the prophylactic administration of Aspirin is not recommended except for other indications and vaginal delivery is not contraindicated. Russo et al. suggest only the use of filtered intravenous lines to prevent air embolism [91]. In pregnant women with PFO and a previous history of thromboembolism, prophylactic low-molecular-weight heparin should be administered during pregnancy and in the first 6–12 weeks post-partum. In pregnant women with PFO and migraine with aura and a previous episode of ischemic attack during migraine, Russo et al. [91] suggest a low dose of aspirin.

In women with a previous stroke related to PFO subjected to transcatheter PFO closure, the pregnancy should be planned after six months of dual antiplatelet therapy. In these patients, low-dose aspirin should be maintained before, during and after pregnancy.

In pregnant women who experience a stroke during pregnancy, the diagnostic flow chart is the same as for other patients, as shown in Figure 1, considering also the May-Thurner syndrome due to an anatomic defect in which the right iliac artery thrombosis of the left iliac vein against the spine provokes a thrombosis of the iliac vein, which may be the cause of paradoxical embolism through a PFO. This condition is rare and often latent but could develop during pregnancy because of uterine growth. The latest American Guidelines [92] do not recommend PFO closure in pregnancy but suggest treating patients with low-dose aspirin or LMWH and postponing closure after delivery. PFO closure in secondary prevention during pregnancy has been performed in cases of high recurrence risk (second episode) or in cases of absolute contraindication to antithrombotic therapy, performed after the 18th week of gestation under exclusive echocardiographic guidance to achieve zero fetal radiation exposure [85].

Prophylactic percutaneous closure is strictly contraindicated during pregnancy and is reserved only for rare, life-threatening maternal-fetal hypoxemia, performed after the 18th week of gestation under exclusive echocardiographic guidance to achieve zero fetal radiation exposure [85].

### 5.2. Neurosurgical Procedures in the Sitting Position

During neurosurgical interventions conducted in the sitting position, a negative hydrostatic pressure gradient is established between the elevated surgical site and the open intracranial venous sinuses, creating an environment highly susceptible to Venous Air Embolism (VAE) [93].

In patients harboring a PFO, a co-existing VAE can rapidly induce acute pulmonary hypertension and a subsequent spike in RAP. This forces the interatrial flap open, triggering a massive, paradoxical air embolism into the cerebral and coronary circulations, which results in catastrophic neurological and cardiovascular complications in up to 14% of cases [93].

Consequently, the sitting position is considered a formal contraindication in patients with a known PFO, dictating systematic preoperative screening [93]. If a sitting approach is deemed mandatory by the neurosurgical team, the current literature suggests performing percutaneous PFO closure 2 to 4 weeks prior to the scheduled surgery [94].

### 5.3. Laparoscopic Surgery and Non-Cardiac Surgical (NCS) Triggers

A preoperatively diagnosed PFO is independently associated with a 2.66-fold increased relative risk of perioperative ischemic stroke within 30 days of NCS under general anesthesia, displaying a distinct embolic architecture with a 3.14-fold higher risk of large-vessel territory strokes [95]. This thromboembolic vulnerability is not restricted to the immediate postoperative period; it extends up to 1 and 2 years post-surgery, driven by sustained systemic inflammation and subclinical deep venous micro-thrombosis following major orthopedic and pelvic oncological surgeries [96].

**Figure 4:**
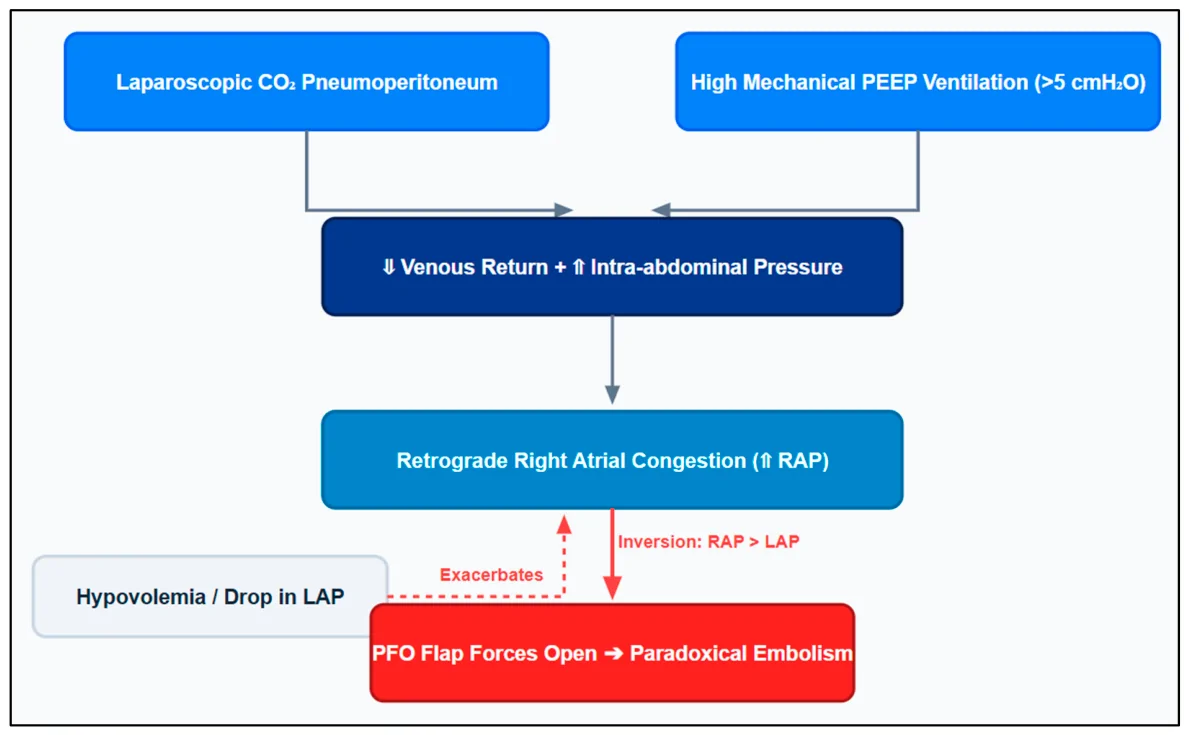
Pathophysiology of air embolism via PFO in laparoscopic surgery. The arrow up 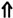 means an increase, while the arrow down 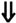 means a reduction.

Laparoscopic interventions introduce unique, intrinsic mechanical challenges that act as intraoperative triggers favoring a right-to-left interatrial pressure gradient (Figure 4).

The induction of a pneumoperitoneum via carbon dioxide (CO_2_) insufflation causes a rapid increase in intra-abdominal pressure that compresses the splanchnic and abdominal venous beds, inducing retrograde venous congestion and acutely driving up RAP [97]. Systemic CO_2_ embolization occurs in approximately 0.15% of standard laparoscopic cases, but this incidence increases to 1.2–4.6% during advanced laparoscopic hepatectomies or nephrectomies, resulting in paradoxical gas embolism to the brain through a patent PFO [97,98,99].

To prevent this iatrogenic pressure shift from forcing open the PFO flap, anesthetic management must adhere to rigorous criteria:

- Intravascular Volume Optimization: Meticulous fluid management is mandatory to avoid any degree of intraoperative hypovolemia or anesthesia-induced vasodilation. A drop in preload reduces left atrial filling pressure (LAP); if LAP falls while RAP is elevated by the pneumoperitoneum, the PFO valve will be held open [99,100].
- PEEP Management: Mechanical ventilation parameters must be tightly regulated, keeping the Positive End-Expiratory Pressure (PEEP) at minimal levels, strictly under 5 cmH_2_O. High PEEP levels of 5 cmH_2_O increase intrathoracic pressure and right ventricular afterload, artificially elevating RAP and promoting a right-to-left shunt for both peripheral microthrombi and insufflated gas [97].
- Venous Thromboembolism Prophylaxis: In the postoperative phase, the early implementation of both mechanical (graduated compression stockings, intermittent pneumatic compression) and pharmacological (LMWH) VTE prophylaxis is mandatory, tailored carefully to the patient’s specific bleeding risk [2].

### 5.4. Proposed Preoperative Risk-Stratification Model for Asymptomatic PFO

According to European consensus recommendations, systematic preoperative screening for PFO in asymptomatic patients undergoing NCS is not indicated (except for sitting-position neurosurgery) [2]. Nakayama et al. [101] retrospectively identified some anatomical features associated with higher risk of paradoxical embolism, such as length of tunnel greater than 10 mm, hypermobility of interatrial septum, a prominent Eustachian valve and massive shunt at TCD (Table 4).

**Figure 5:**
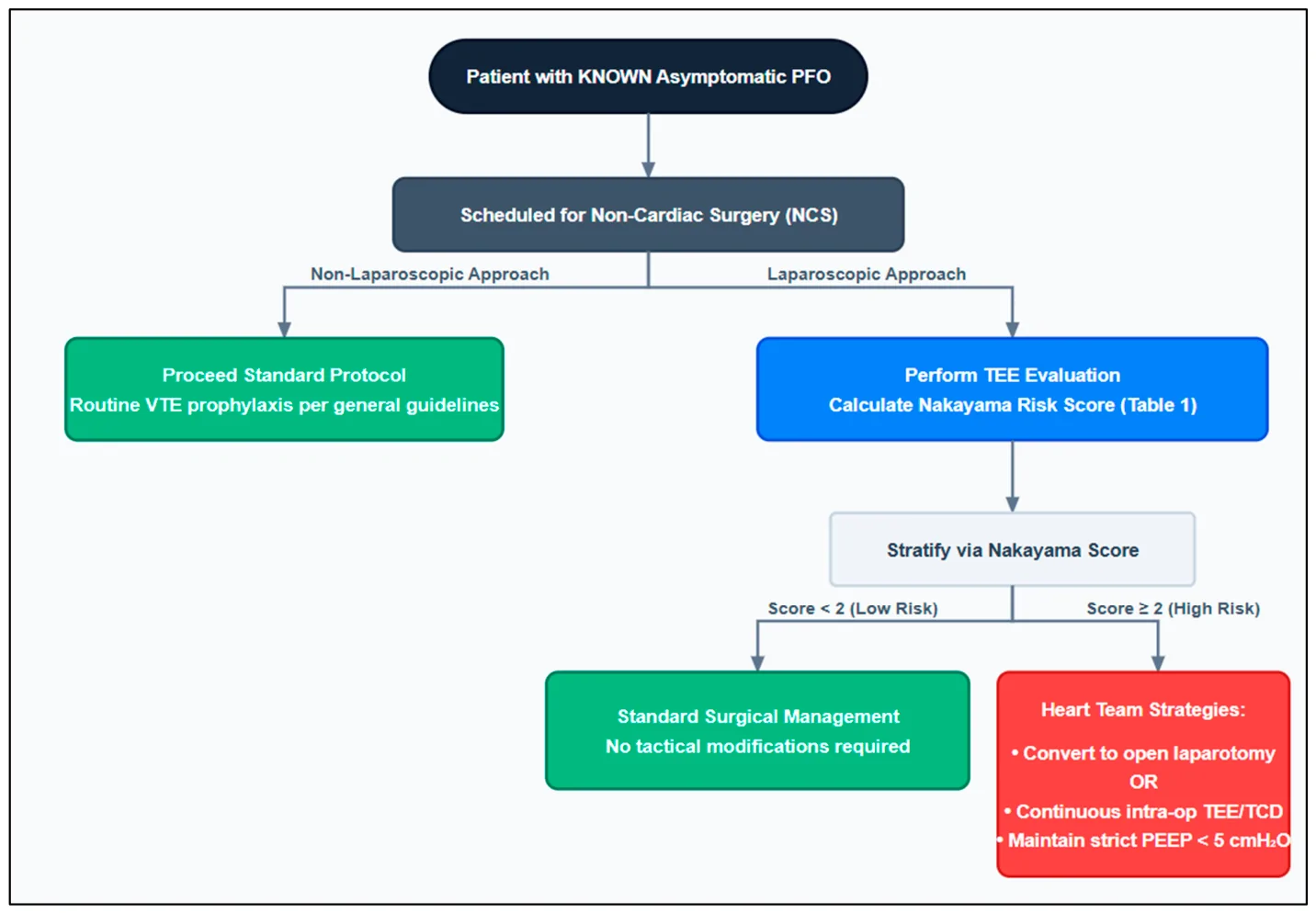
Perioperative management algorithm for known asymptomatic PFO.

An Italian study group [97] proposed a stratification score system of patients with asymptomatic PFO planned for non-cardiac surgery using the Nakayama criteria to define the PFO as high or low risk. A total score ≥ 2 identifies a PFO anatomy at high risk for embolic events [101,102]. Based on this score, a clinical algorithm is proposed for the perioperative management of patients with a known, asymptomatic PFO who are candidates for laparoscopic surgery (Figure 5).

In patients presenting with a score ≥ 2, management must be thoroughly discussed within a multidisciplinary Heart Team. The option to perform a prophylactic percutaneous PFO closure prior to non-cardiac surgery is not supported by high-level clinical evidence [2]. The proposed stratification model shown in Figure 5 is not recommended by more recent guidelines [7,9] and could be prospectively validated in future trials. The device closure option should only be considered in highly selected cases that combine a high-risk anatomy with major pelvic oncological or complex orthopedic surgeries that carry an extreme thromboembolic burden, balancing procedural risks against the necessity of perioperative dual antiplatelet therapy (DAPT) [2]. A meta-analysis by Hobbes et al. supported the increased perioperative stroke risk associated with PFO but found no evidence that PFO directly increased long-term adverse outcomes in perioperative strokes [102].

## 6. Role of PFO in Pediatric Population

The prevalence of PFO or atrial septal defect has been reported as approximately 0.1–0.2% at birth, with approximately 40% of them spontaneously closing in the first 12 months of life or in the first years of childhood [103]. In children, the diagnosis of PFO is an incidental finding during echocardiography and should not be considered a disease except for cases of patients with ESUS or transient ischemic attack or migraine with aura refractory to standard therapy.

In pediatrics, most arterial ischemic strokes and transient ischemic attacks are associated with prothrombotic disorders or arteriopathy. Paradoxical embolism through a PFO is an exclusion diagnosis in this population, and the closure of PFO is not recommended when the stroke or acute ischemic attack is not associated with a higher risk of thromboembolism [104].

Recent guidelines [8] on congenital heart disease lack clear recommendations in this subset of patients because very few studies have been published in the pediatric population and the majority of them regard stroke or migraine with aura. Only one case report has been published in a child affected by platypnea-orthodeoxia syndrome [105].

In a retrospective study of 95 young patients [106] subjected to transcatheter PFO closure for migraine or dizziness, an improvement in symptoms was observed (90% of patients) and the resolution of migraine or dizziness in 24% of patients, demonstrating a promising clinical benefit, but further clinical trials are necessary to provide recommendations.

In asymptomatic children, the presence of PFO is a benign condition not requiring therapy or particular follow-up. An interesting multicenter observational study [107] demonstrated the absence of correlation with unexplained syncope, palpitations or chest pain. Conversely, an atrial septal defect is a different condition caused by incomplete growth of the interatrial septum during embryogenesis. It can be associated with a significant L-to-R shunt and consequently a right ventricular volume overload. In this case, the closure of the ASD is mandatory and is well defined in the congenital heart disease guidelines [8].

## 7. Conclusions

PFO is a benign, highly prevalent condition, but some PFOs have a major risk of complications when associated with specific anatomic features, such as ASA, severe R-to-L shunt, large tunnel, prominent Eustachian valve. To date, the strongest evidence regards the link between PFO and ESUS, for which a decisional algorithm has been proposed and validated. However, in rare conditions, such as migraine with aura, desaturation syndrome and decompression sickness, the decision to search for and eventually treat the PFO should be made on a case-by-case basis by a multidisciplinary team involving various specialists—such as the cardiologist, the neurologist and the neuro-radiologist—hoping for future production of more evidence in these subgroups of patients. The transcatheter closure is to date a widespread, safe and effective procedure, with a low incidence of complications in both short- and long-term follow-up. Migration and atrial or aortic erosion are the worst complications but have a low incidence, which could be further reduced with the development of smaller and less rigid prostheses.

## Figures and Tables

**Table 1:** RoPE: risk of paradoxical embolism.

Category	Details	Points
No History of	HTN	+1
	Diabetes	+1
	Stroke/TIA	+1
	Smoke	+1
Cortical Infarct	MRI/CT	+1
Age	18–29	+5
	30–39	+4
	40–49	+3
	50–59	+2
	60–69	+1
	≥70	0

HTN: hypertension; TIA: transient ischemic attack; MRI/CT: magnetic resonance/computed tomography.

**Table 2:** RoPE result and estimation of PFO probability.

Pts: 0–3	Low probability
	(0–38%)
Pts: 4–6	Moderate probability
	(34–62%)
Pts: 7–10	High probability
	(72–88%)

**Table 3:** High-risk PFO features.

A	Large shunt size *
B	ASA **

ASA: atrial septum aneurysm; * >20 bubbles in the left atrium on TOE (transesophageal echocardiography); ** >10 mm of excursion from midline.

**Table 4:** Anatomical risk scoring system for PFO embolism.

Echocardiographic Parameter (TEE)	Anatomic Criterion	Points
PFO Tunnel Length	>10 mm	1
Atrial Septal Aneurysm (ASA)	Total excursion 10 mm or hypermobility	1
Eustachian Valve/Chiari Network	Prominent, directing IVC flow toward the PFO	1
Resting Shunt Size (Baseline)	Massive > 20 microbubbles in the left atrium	1

(Modified from Nakayama et al.) [101].

## Data Availability

No new data were created or analyzed in this study. Data sharing is not applicable to this article.
